# A Transcriptome Response of Bread Wheat (*Triticum aestivum* L.) to a 5B Chromosome Substitution from Wild Emmer

**DOI:** 10.3390/plants13111514

**Published:** 2024-05-30

**Authors:** Alexandr Muterko, Antonina Kiseleva, Elena Salina

**Affiliations:** Institute of Cytology and Genetics SB RAS, 10 Akad. Lavrentyeva Avenue, Novosibirsk 630090, Russia

**Keywords:** chromosome substitution, transcriptome, wild emmer, wheat

## Abstract

Over the years, alien chromosome substitution has attracted the attention of geneticists and breeders as a rich source of remarkable genetic diversity for improvement in narrowly adapted wheat cultivars. One of the problems encountered along this way is the coadaptation and realization of the genome of common wheat against the background of the introduced genes. Here, using RNA-Seq, we assessed a transcriptome response of hexaploid wheat *Triticum aestivum* L. (cultivar Chinese Spring) to a 5B chromosome substitution with its homolog from wild emmer (tetraploid wheat *T. dicoccoides* Koern) and discuss how complete the physiological compensation for this alien chromatin introgression is. The main signature of the transcriptome in the substituted line was a sharp significant drop of activity before the beginning of the photoperiod with a gradual increase up to overexpression in the middle of the night. The differential expression altered almost all biological processes and pathways tested. Because in most cases, the differential expression or its fold change were modest, and this was only a small proportion of the expressed transcriptome, the physiological compensation of the 5B chromosome substitution in common wheat seemed overall satisfactory, albeit not completely. No over- or under-representation of differential gene expression was found in specific chromosomes, implying that local structural changes in the genome can trigger a global transcriptome response.

## 1. Introduction

Wheat is one of the most important crops for human consumption, with a substantial role in global food production [1]. Because it is one of major sources of carbohydrates and protein in the human diet, the yield and nutritional quality of this crop have a considerable impact on human health and wellbeing. Continuous changes in the condition of the environment, in the climate, and phytopathogen evolution pose a challenge for wheat production and utilization and continuous improvement of narrowly adapted wheat cultivars is required.

Human civilization and domestication of wheat have evolved simultaneously. Upon domestication, however, the initial large gene diversity observed in wild progenitors has passed through two major genetic bottlenecks [2,3] and diminished dramatically in traditional varieties of bread wheat [4,5,6]. Continued loss of allelic diversity in modern cultivars can make global wheat production increasingly vulnerable to abiotic stressors and new diseases. As a rich source of remarkable genetic diversity with a large number of morphological, agronomic, and biochemical traits, less cultured and wild wheat taxa have been used for decades in the breeding of common wheat (*Triticum aestivum* L., AABBDD genome, 2n = 42) [7].

Wild emmer wheat *Triticum turgidum* ssp. *dicoccoides* Koern (AABB genome, 2n = 28) is one of progenitors of cultivated wheat and has had a core role in the gradual multisite wheat domestication [8,9]. Because of the occurrence of homologous recombination between the A and B genomes of different wheat species, wild emmer is capable of producing fertile offspring with tetraploid and hexaploid wheat cultivars [10], allowing for gene introgression for wheat improvement. For decades, actual potential and genetic resources of wild emmer for wheat breeding have been a subject of numerous studies. The gene pool of *T. dicoccoides* is recognized as a useful reservoir of valuable genetic material for various agronomically important quantitative and qualitative traits such as earliness, yield, the amount and desirable composition of grain protein, tolerance to abiotic stressors, and resistance to fungal diseases and wheat mosaic virus, and underlies much of the variation in morphological traits (reviewed in [11,12,13,14]). Despite its highly promising potential to improve the narrow gene pool of modern wheat cultivars, wild emmer has not been very widely and fully exploited in wheat breeding and improvement. The reason is the lack of theoretical knowledge and some unsolved practical agronomic problems. One of such problems is the coadaptation and realization of the genome of bread wheat against the background of introduced alien genes or their linked groups owing to evolutionary divergence of *Triticum* species.

Over the years, chromosome 5B of common wheat has attracted the attention of wheat geneticists and breeders. This chromosome has unique morphology with the greatest difference in arm lengths from other chromosomes of B, A, and D genomes [15]. It carries genes of numerous agronomically important traits, including but not limited to the vernalization requirement, microelements’ uptake, hybridic necrosis, crossability with rye, preharvest sprouting, a hair peduncle, the grain protein content, and resistance to phytopathogens [16]. Specific activity of some genes (*Ph1*) in 5B suppresses both homoeologous pairing [17,18] and pairing of differentiated homologs [19] and hence controls and supports bivalent conjugation of chromosomes in hexaploid wheat. This feature has a pivotal role in the mechanism of cytological diploidization of polyploid wheat and ensures good potential for high fertility and genetic stability, which have caused this species to become widespread throughout the world. In due time, this feature has also helped to clarify many issues regarding the origin and evolution of bread wheat and allowed breeders to design some techniques for its improvement.

Due to large genetic stocks of aneuploid lines, sets of tag sequences, and other resources, the common wheat variety Chinese Spring (CS) is often the focus of wheat genetic and cytogenetic studies. This landrace was the first one chosen for genome sequencing of hexaploid wheat [20]. Germplasm of CS lines substituted with single chromosomes of wild emmer *T. dicoccoides* is available (USDA-ARS, https://wheat.pw.usda.gov/GG3/germplasm, accessed on 20 March 2024). Despite long-term research on alien substituted lines of polyploid wheat, their transcriptome has not been investigated in a comprehensive manner. A possible reason is the absence (until recently) of a reference genome of hexaploid wheat as well as difficulties with quantification of RNA-Seq data after gene introgression into polyploid wheat [21].

To our knowledge, the present study is the first report on a transcriptome response of common wheat to a substituted chromosome from wild emmer. In particular, we used an alien substitution line (DS) of CS carrying chromosome 5B of wild emmer (tetraploid wheat *T. dicoccoides*). In our previous work on this substituted line, rearrangements in 5B primary structure between these wheat species were identified, which were assumed to affect suppression of homologous recombination in some regions of short and long arms of this chromosome [22]. Here, after assessing the transcriptome response by the RNA-Seq technique, we discuss how complete the physiological compensation for this alien chromosome substitution is.

## 2. Results

### 2.1. Transcriptome Quantification

The reference transcriptome consisted of 265,416 sequences partitioned into 118,804 clusters of paralogous genes. The RNA-Seq libraries included an average of 33 million reads; among them, 10–30% were completely and uniquely mapped to the reference transcriptome. Assuming that fragments of the same transcript do not overlap, the transcriptional abundance of genes was quantified by means of the greatest coverage depth, which we believe makes the quantification more reliable and independent from transcript length. The counts between libraries were normalized to the level of *MscS* from chromosome 5A; this is a housekeeping gene with stable expression under different conditions in different wheat tissues [23]. Both the mean and median values of the size factor across samples, as calculated from the normalized counts under the assumption that the majority of genes are expressed nondifferentially [24], were 1.0, confirming that the choice of the aforementioned housekeeping gene was appropriate for library normalization.

Instead of calculating the mean counts across replicates using the method of moments, they were estimated by fitting of a negative binomial distribution (NBD), which characterized the observed distribution of counts across replicates the best. During this procedure, in 94% of 1,006,232 cases, the mean was estimated using all 3 replicates; in 6% of the cases, 1 of the replicates was rejected, and in 494 cases (<0.05%), the genes were excluded from the analysis in at least 1 of samples because the dispersion among replicates was greater than the allowed 1. The mean values estimated in this way are believed to be safer from outliers.

Under the tested conditions and at the developmental stage, the overall expression magnitude of the transcriptome was low in both lines. Out of the 265,416 genes of the reference transcriptome, expression of 125,779 was quantified, and only for 54,728 (43.5%) of them, the estimated mean count was at least 8 at least in 1 of the samples, whereas most of genes of the reference transcriptome (~80%) were inactive, expressed at very low levels, or were not quantified because they were covered exactly by multiple mapped reads or no reads were mapped.

### 2.2. Diurnal Transcriptome Activity

**Figure 1:**
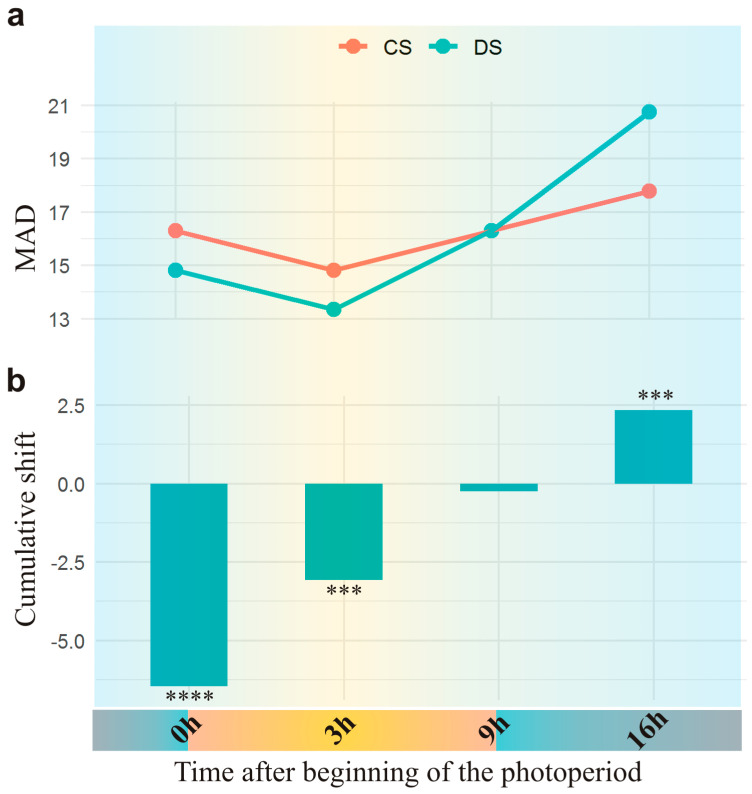
Diurnal transcriptome activity. (**a**) The transcriptome expression level across the time points in Chinese Spring (CS) and substituted line (DS) is presented as median absolute deviation (MAD) of estimated mean counts of 54,346 genes. (**b**) The cumulative shift of count distributions between the lines, as estimated across the time points. Statistical significance of this difference in the transcriptome expression level between the lines is indicated: *** *p* < 0.001,**** *p* < 1 × 10^−4^.

The trends of expression dynamics across time points for 54,346 genes were similar between the two lines (Figure 1a). This analysis showed a decrease in general transcription activity after the beginning of the photoperiod until time point 3 h (more pronounced in CS) and a subsequent gradual increase until 16 h. Although the rate of this increase in transcription activity was higher in DS than in CS, it was similar between the two lines from 3 h to 16 h. Notably, although in CS, transcriptome activity did not change during the night (a shift of transcriptome activity between 16 h and 0 h was +0.005, *p* > 0.99), in DS, it dropped sharply and significantly between 16 h and 0 h (shift = −8.8, *p* < 1 × 10^−4^).

The cumulative shift of count distributions between CS and DS was negative at the first three time points, indicating a decrease in transcription activity in DS at the beginning of and during the light phase of the photoperiod (Figure 1b). Although this transcriptome underexpression in DS was the most pronounced at 0 h (shift = −6.5, *p* < 1 × 10^−4^), its activity rose more quickly than that in CS, thereby achieving a similar level in both lines at the end of the light phase, i.e., at 9 h (shift = −0.24, *p* > 0.75), and led to some overexpression of the DS transcriptome in the middle of the night, i.e., at 16 h from the beginning of the photoperiod (shift = +2.3, *p* = 0.001).

The gene pair correlation analysis (997,280,130 pairs of 44,661 genes for each line) revealed similar proportions of coexpressed genes when the two lines were compared but slightly more in CS (1,953,807 gene pairs) than in DS (1,627,424 gene pairs). The proportion of paralogs among coexpressed genes was 0.51–0.53 per gene: much less than 2.75 in the tested gene set or the expected 2 paralogs per gene for hexaploid wheat. This finding implies less than 50% of common variance (*r* < 0.7) between the transcription profiles of most of paralogous (including homoeologous) genes among the 31,838 tested (a subset of 44,661 genes paired with at least one paralog). The preservation rate of coexpression across specimens was only 24.3%. Out of these 475,555 gene pairs, for 421, the coexpression got preserved while the diurnal expression pattern drastically transformed between lines (*r* < 0.5), implying strong coregulation between genes of these pairs. Only eight pairs consisted of paralogous genes (0.075 paralogs per gene), assuming that coregulation is not an expected attribute of homoeologous genes, at least in this case. Time point correlation analysis of the gene pairs coexpressed in CS, but not in DS, detected a change in expression at 9 h as a major contributor to the loss of coexpression in DS (*r* = 0.32), whereas the highest covariance in expression between these genes was observed at 3 h (*r* = 0.42) and 16 h (*r* = 0.39). Pairwise correlations in diurnal gene expression confirmed the loss of correlation between the lines (*r* < 0.7) for most of the transcriptome (93.4%, 41,699 genes), implying a global genomic effect of the 5B chromosome substitution.

The variance of expression was estimated for 54,346 genes at each time point, by means of specific deviations from the mean diurnal expression of each gene, to assess the effect of the 5B chromosome substitution on the change in diurnal transcriptome activity. In CS, this variance was the highest at 3 h and could be ranked as 3 h > 0 h > 9 h = 16 h, whereas in DS, the highest variance was at 16 h, and the ranking was inverse (16 h > 9 h > 0 h = 3 h). This finding implies inversion of the highest and lowest deviation from the mean diurnal expression between the middle of the light phase and the middle of the dark phase under the influence of the shift of the mean diurnal expression until the beginning of the photoperiod in DS. Analysis of the cumulative shift of the distributions of the unsigned deviation from the mean diurnal expression between the specimens confirmed less deviation at 0 h and 3 h and more deviation at 16 h in DS (*p* < 1 × 10^−4^). This inversion of transcriptome activity perhaps occurred at 9 h, because at this time point, the difference between lines in the deviation distribution was not significant (*p* > 0.39). The variance was also analyzed via particular deviations from the mean diurnal expression across all samples. The obtained distribution of variance was consistent with the overall transcriptome expression level, thereby confirming a relative decrease in variance at 0 h and its gradual increase until 16 h in DS, whereas in CS, the trend was opposite.

### 2.3. Differential Gene Expression Analysis

A total of 7789 genes were found to be expressed differentially with unsigned log_2_FC of at least 0.9 (Table S1). The largest number of differentially expressed genes (DEGs) (3323) was identified at 0 h, and this number was ~1.5 times greater than that at any other time point, whereas the largest number of genes with the greatest fold change and expression level was detected at the 3 h time point (402 genes). Overall, most of DEGs were expressed weakly or the difference in expression between specimens was not large. Sixteen genes showed differential expression across all time points. For nine of them, the correlation between patterns of diurnal expression in CS and DS persisted during the whole experiment (*r* > 0.73), indicating a stable change in amplitude of their expression during the experiment (one gene was upregulated and eight genes were downregulated at all time points). The loss of correlation between the lines (*r* < 0.7) under the influence of differential expression was confirmed for 6643 (85%) of DEGs.

**Figure 2:**
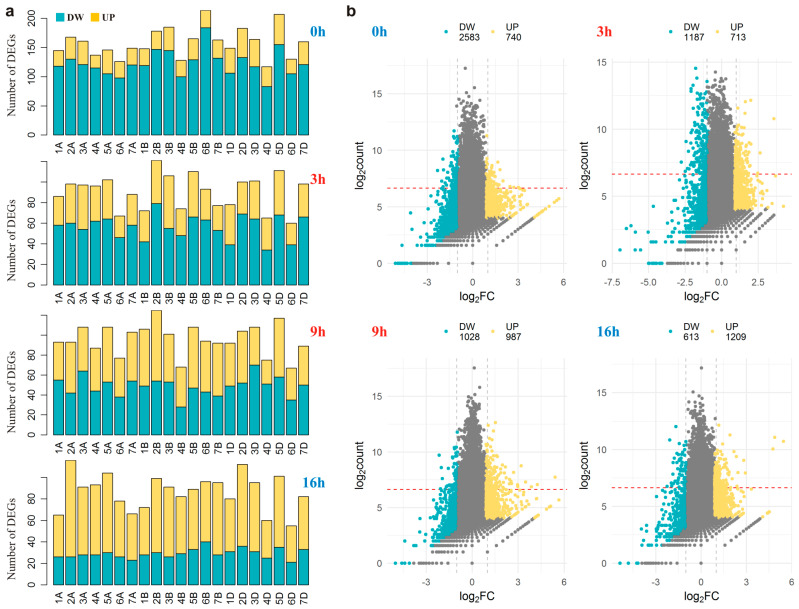
Differential gene expression in the substituted line. (**a**) The distribution of differentially expressed genes (DEGs) across the genome at each time point. The number of up-(UP) and downregulated (DW) genes is indicated for each chromosome. (**b**) Scatter plots of log_2_ fold change for each gene versus log_2_ estimated mean counts. The differential expression that did not reach statistical significance is highlighted in gray. The red dashed line indicates the threshold at which gene expression was assumed to be high enough for a pronounced physiological response (at least half of expression of an endogenous control: the *MScS-5A* gene).

The DEGs were almost uniformly distributed across the genome (Figure 2a), from 265 per chromosome (4D chromosome) to 453 (2B chromosome). Thus, most of DEGs were located on chromosomes other than 5B (95% DEGs) or outside the homeologous 5 group (84% DEGs), implying a global impact of the alien chromosome substitution on the transcriptional landscape of the CS genome. This finding also makes read mismapping and incorrect assessment of the homeolog expression balance owing to the loss of similarity between chromosomes 5B of CS and DS unlikely, which can occur during transcriptome quantification [21].

According to sequence similarity, all DEGs were partitioned into 1022 clusters containing two or more paralogs and 5316 of nonparalogous genes. Nonetheless, half of the clusters of paralogous DEGs did not confirm a similar direction of good-confidence log_2_FC (|log_2_FC| ≥ 0.9) for all paralogs of a cluster at any time point, indicating lack of strong coregulation of paralogs (homeologs), as was evident from the general transcriptome analysis above, for most of DEGs.

The average UP/DOWN ratio of 0.67 pointed to the predominance of downregulation in the substituted line. Nevertheless, the distribution of up- and downregulated genes differed across the time points (Figure 2b). Although before the beginning of the photoperiod (0 h), the downregulated genes dominated by threefold (UP/DOWN = 0.29), during the experiment, the proportion of upregulated genes gradually increased, such that at the last time point (16 h), i.e., 7 h after the end of the photoperiod, upregulated genes predominated by twofold (UP/DOWN = 1.97). This trend was confirmed in the substituted line at both the whole-genome level and chromosomal level (Figure 2a) and was in agreement with the diurnal transcriptome activity described above (Figure 1a).

The effect of differential expression on a change in gene activity was assessed next. Only 22% of 9060 cases of differential expression were related to a change in amplitude of expression that enhanced current gene activity (over- or underexpression relative to the mean diurnal expression in CS). In the other cases, differential expression was accompanied by weakening (32%) or inversion of gene activity (passing through the mean diurnal expression in DS), which predominated (67%). In 35 cases, the inversion of diurnal gene activity was inadequate to change the differential-expression status (UP/DOWN), indicating a shift of the mean diurnal expression in DS under the influence of differential expression at other time points. These results suggested that in wheat, the 5B chromosome substitution accompanied by differential expression can cause drastic changes in diurnal gene activity, up to its inversion in most cases.

### 2.4. Functional Annotation of DEGs

**Figure 3:**
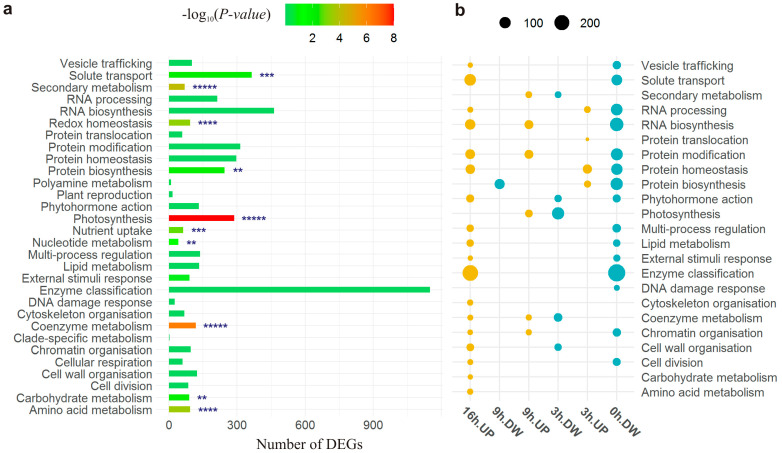
Functional annotation of differentially expressed genes (DEGs) in the substituted line. (**a**) Biological processes annotated in MERC. Statistical significance of over-representation is indicated:** *p* < 0.01, *** *p* < 0.001, **** *p* < 1 × 10^−4^, ***** *p* < 1 × 10^−5^. Values of −log_10_(*p*-value) greater than 8 were floored to 8. (**b**) Across time points, over-representation of up- (UP) and downregulated (DW) DEGs in the substituted line.

During gene ontology enrichment analysis, functional annotations in terms of BPs were assigned to 3813 (49%) DEGs (Figure 3a). They included 30 BPs out of the 30 tested BPs (Table S2), suggesting that metabolism as a whole was affected by the differential expression. For 10 BPs, over-representation was noted (*p* < 0.012), with the highest significance for “Photosynthesis” (288 DEGs, *p* < 1.8 × 10^−39^), but the distribution of these BPs differed across the time points (Table S3). In particular, although “Photosynthesis” was over-represented at all time points, most other BPs were over-represented only at certain time points. Specifically, the highest significance of over-representation was obtained at 0 h for “Photosynthesis” (77 DEGs, *p* < 1.8 × 10^−4^) and “Protein biosynthesis” (140 DEGs, *p* < 1.6 × 10^−8^), at 3 h for “Photosynthesis” (129 DEGs, *p* < 3.3 × 10^−36^) and “Coenzyme metabolism” (50 DEGs, *p* < 4.9 × 10^−8^), at 9 h for “Photosynthesis” (74 DEGs, *p* < 8.5 × 10^−9^) and “Amino acid metabolism” (39 DEGs, *p* < 1.4 × 10^−5^), and at 16 h for “Photosynthesis” (92 DEGs, *p* < 1.8 × 10^−19^) and “Solute transport” (133 DEGs, *p* < 2.8 × 10^−9^).

For 23 BPs, over-representation among up- or downregulated genes at least at one time point was detected (Figure 3b). Although these BPs had similar ratios of DEGs (UP/DOWN = 28/21), their distribution was not uniform across the time points. Their largest numbers were identified at 0 h (31%) and 16 h (37%). At these time points, regardless of BPs, there were exclusively down- and upregulated genes, respectively (Figure 3b). Same BPs showed over-representation of up- and downregulation at two or more time points. Most of these BPs were shared by points 0 h and 16 h. In particular, for 13 of the 20 BPs over-represented by a differential expression type at 0 h or 16 h, DEGs were downregulated at 0 h and upregulated at 16 h, in agreement with the above-mentioned general trend of the expression of DEGs in DS. Moreover, for each BP, most of these genes were not shared between the time points, indicating that mostly different DEGs (involved in the same BPs) were turned “off” and “on” before the beginning of the light phase and in the middle of the dark phase of the photoperiod, respectively.

Downregulation was over-represented among the DEGs related to “Photosynthesis” at 3 h (*p* < 3.7 × 10^−3^), whereas upregulation was over-represented among genes associated with this BP at the 9 h time point (*p* < 5.5 × 10^−9^) (Figure 3b), suggesting that although in CS, “Photosynthesis” was upregulated at 3 h, in DS, it was downregulated but upregulated at the next, 9 h, time point. This downregulation of “Photosynthesis”-related genes in DS at 3 h was more pronounced than their upregulation at 9 h. In particular, although “Photosynthesis” was associated with 129 DEGs at 3 h, 120 of them (93%) were downregulated, whereas at 9 h, 74 DEGs were related to “Photosynthesis”, and only 42% of them were upregulated. The test for over-representation of BPs among the up- and downregulated DEGs across time points confirmed over-representation of “Photosynthesis” in the set of upregulated genes at 9 h (*p* < 4.1 × 10^−5^) and in the downregulated DEG set at 3 h (*p* < 5.8 × 10^−8^) and 16 h (*p* < 4.1 × 10^−15^). Taken together, these results point to a possible delaying effect of the chromosome 5B substitution on activation of “Photosynthesis”, as follows from the downregulation of relevant genes at the beginning of the light phase of the photoperiod and upregulation at the end.

**Figure 4:**
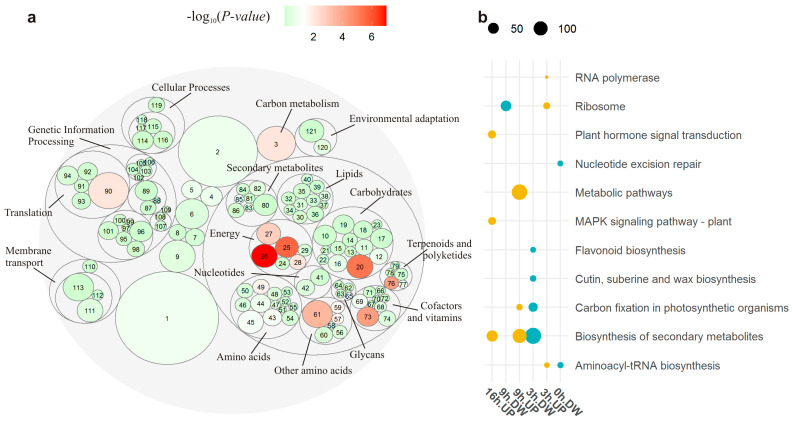
Pathways containing differentially expressed genes (DEGs) in the substituted line. (**a**) Pathways annotated by means of KEGG. The main class of nested pathways is indicated. The numbers correspond to the pathways listed in Table S4. Values of –log_10_(*p*-value) greater than 7 were floored to 7. (**b**) Across time points, over-representation of up- (UP) and downregulated (DW) DEGs in the substituted line.

A total of 135 out of 143 tested KEGG pathways were associated with DEGs (Table S4, Figure 4a). Among the most DEG-rich (>200 DEGs) metabolic pathways, there were “Biosynthesis of secondary metabolites” (522 DEGs), “Amino acid metabolism” (421 DEGs), “Carbohydrate metabolism” (372 DEGs), and “Energy metabolism” (202 DEGs). Among other pathways, there was “Translation” (251 DEGs) and particularly “Ribosome” (134 DEGs). For 13 pathways, statistically significant over-representation was shown (Figure 4a), confirming the highest significance of “Photosynthesis” (44 DEGs, *p* < 2.4 × 10^−6^) and especially “Photosynthesis—antenna proteins” (56 DEGs, *p* < 8.7 × 10^−30^). Among pathways abundant with DEGs, “Ribosome” and “Carbon metabolism” were over-represented too (*p* < 8.5 × 10^−3^). In terms of time points, the test for over-representation of pathways in DEG sets (Table S5) found “Ribosome” alone at 0 h (*p* < 1.3 × 10^−6^), whereas the “Photosynthesis—antenna proteins” pathway was over-represented in each of next three time points (*p* < 1.2 × 10^−10^, 5.4 × 10^−14^, and 3.4 × 10^−19^, respectively). This finding was consistent with the overall pattern of over-representation of pathways among DEGs, implying that “Photosynthesis” (energy metabolism) and “Ribosome” (translation) pathways were the most affected by differential expression in the substituted line.

In terms of time points, the test for over-representation of up- and downregulated DEGs confirmed the predominance of downregulated pathways in the substituted line mainly at 0 h and 3 h but not at 16 h (Figure 4b). In contrast, none of pathways over-represented in the upregulated DEG set were found at 0 h (Figure 4b). Downregulation of “Carbon fixation” and “Biosynthesis of secondary metabolites” was over-represented at 3 h (*p* < 2.3 × 10^−2^, and *p* < 1.4 × 10^−4^), but upregulation of these pathways was over-represented at 9 h (*p* < 4.1 × 10^−4^, and *p* < 1.9 × 10^−3^). A small number of upregulated DEGs was statistically significantly associated with “Genetic information processing” pathways [“RNA polymerase” (three DEGs, *p* < 3.9 × 10^−2^), “Aminoacyl-tRNA biosynthesis” (seven DEGs, *p* < 1.7 × 10^−2^), and “Ribosome” (14 DEGs, *p* < 4.1 × 10^−4^)] at 3 h. On the other hand, downregulation of 47 DEGs associated with the “Ribosome” pathway was well pronounced at 9 h (*p* < 2.6 × 10^−3^).

### 2.5. Differential Activity of BPs and Pathways

**Figure 5:**
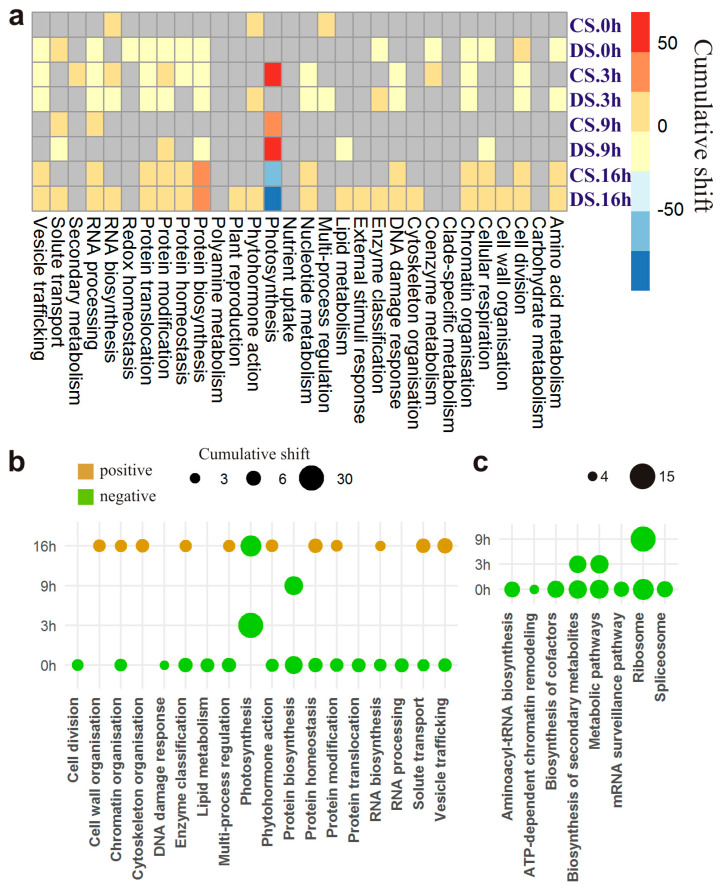
Diurnal and differential activity of biological processes (BPs) and pathways. (**a**) Diurnal activity of BPs in CS and in the line carrying the substituted 5B chromosome (DS). (**b**) Differential activity of BPs between the lines at different time points. (**c**) As in b, but for pathways.

A total of 30 BPs were tested for the significance of change in their diurnal activity after the 5B chromosome substitution. The distribution of relative activity of BPs across time points was overall in agreement with plant cell physiology. In particular, at 16 h, the highest and most significant upregulation was registered for “Protein biosynthesis” (*p* < 1 × 10^−4^) but strong downregulation for “Photosynthesis” (*p* < 1.4 × 10^−3^ and *p* < 1 × 10^−4^ for CS and DS, respectively), which nonetheless turned out to be upregulated at 9 h (*p* < 4.8 × 10^−2^ and *p* < 3.9 × 10^−3^) in both lines (Figure 5a). Most of BPs, except “Photosynthesis”, showed a positive change in activity (were overexpressed) in the dark phase (at 0 h but mainly at 16 h), with the highest and significant upregulation at 16 h in both lines, and declined below their individual mean diurnal expression in the light phase of the photoperiod (Figure 5a). Significance of the change in diurnal activity was below the threshold (*p* ≥ 0.05) at 9 h for most of these BP, suggesting that at this time point, their activity was close to the daily average.

Transcription activity of “Photosynthesis” in CS manifested the opposite trend, with the start of overexpression before the beginning of the photoperiod (0 h, shift = +23.1), a peak near the midday (3 h, shift = +68.0), a decrease at the end of the light phase (9 h, shift = +34.8), and a strong negative change of activity (underexpression) at night (16 h, shift = −57.4). In the substituted line, this “Photosynthesis” activity rhythm, which is canonical for the plant cell, was found to be disrupted. In particular, in addition to a significant drop in expression at 16 h (shift = −99.6), downregulation of its transcription activity was also noted and was similar at 0 h (shift = −10.8) and 3 h (shift = −10.2), whereas upregulation was documented at 9 h (shift = +52.0). While the downregulation of this BP was significant in both lines at 16 h (*p* < 1.4 × 10^−3^ and *p* < 1 × 10^−4^ for CS and DS, respectively), the significance of the upregulation was reached at 3 h only in CS (*p* < 2 × 10^−4^); at 9 h, there was the best significance in DS (*p* < 3.9 × 10^−3^), whereas in CS, significance was marginal (*p* = 4.8 × 10^−2^). These results are consistent with our observed shift of the mean diurnal expression of the transcriptome toward the light phase of the photoperiod in DS as well as confirm the decrease (sometimes even inversion) in activity of “Photosynthesis” at 0 h and 3 h and its overexpression at 9 h in DS, in contrast to CS, during our DEG analysis.

To assess the effect of the chromosome 5B substitution in CS on the change in the pattern of diurnal transcription activity, the significance of differential activity between lines was evaluated for each BP across all time points. For 18 of 30 analyzed BPs, differential activity was found at least at one time point (Figure 5b). Most of the pathways (90%) for which the differential activity between lines was significant were detected in the dark phase of the photoperiod (0 h and 16 h). For all BPs, the fold change in activity was negative at 0 h (underexpression in DS) and positive at 16 h (overexpression in DS), in agreement with the over-representation of up- and downregulated DEGs at corresponding time points (Figure 2b and Figure 4a). Nonetheless, the significance of differential activity for most BPs was higher at 0 h than at 16 h. The differential activity of “Photosynthesis” between CS and DS was significant and negative at 3 h (shift = −30, *p* < 6.7 × 10^−3^) and near the threshold of significance at 16 h (shift = −17, *p* = 0.04) but not at the other time points. These data support the supposition of more reliable downregulation of this BP in the substituted line at 3 h rather than its overexpression at 9 h relative to its activity in CS, as proposed here during the differential-expression analysis.

A total of 53 pathways were assessed regarding differential activity, which was statistically significant only for 8 pathways at least at 1 time point (3 of them at 2 points). In all 11 cases, the activity change was negative (underexpression in DS) and most strongly pronounced for the “Ribosome” pathway at 9 h (shift = −14.7, *p* < 1.2 × 10^−3^). In line with the total transcriptome analysis and differential-expression analysis, all of these pathways were detected at 0 h, and their number diminished gradually over time, such that only two and one underexpressed pathways were found at 3 h and 9 h, respectively, and no pathways with statistically significant differential activity between specimens were found at 16 h (Figure 5c).

### 2.6. Time Point-Associated Expression

To evaluate the influence of the chromosome 5B substitution on preservation of condition-related transcriptome fractions, genes were clustered by means of a sharp change in their diurnal expression at one of the four time points. The eight clusters included strongly (at least a twofold difference from any other time point) upregulated (overexpressed) and downregulated (underexpressed) genes at one of time points, and over-represented BPs were analyzed. A total of 5812 and 6078 genes were clustered in CS and DS, respectively. Among overexpressed clusters, in both lines, the cluster of genes overexpressed at 3 h contained fewer genes, whereas genes strongly downregulated at this time point formed the largest cluster in CS and to a lesser degree in DS. Overall, the size of clusters containing overexpressed genes was in agreement with the diurnal pattern of the transcriptome expression level in these specimens and with the distribution of up- and downregulated DEGs, so that clusters overexpressed at 0 h and 3 h were twice as large in CS, those at 16 h were twice as large in DS, and the cluster containing overexpressed genes at 9 h had a size similar between the two lines but was slightly larger in DS (DS/CS = 1.16). In both lines, the size of underexpressed clusters was overall inversely related to the size of corresponding overexpressed clusters.

**Figure 6:**
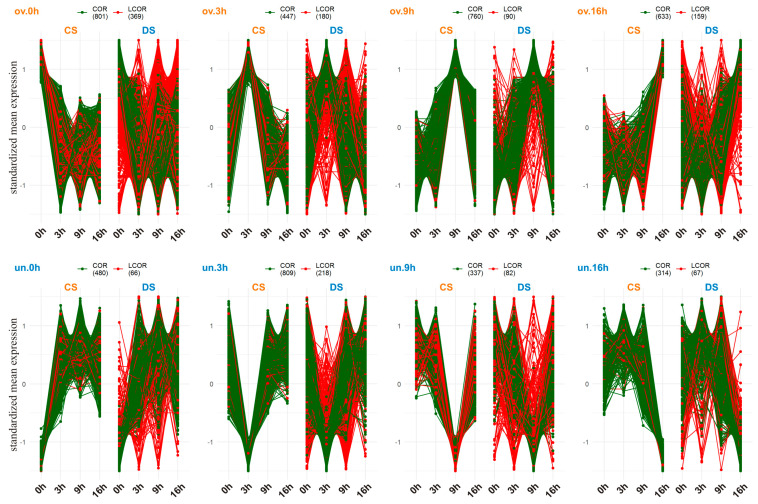
Diurnal expression of genes that were overexpressed (ov) or underexpressed (un) at a certain time point in CS is presented for both lines. Genes with correlated diurnal expression between the lines (COR) are highlighted in green, whereas the loss of this correlation (LCOR, *r* < 0.7) is shown in red.

The preservation of CS clusters in DS was in the range of 25–60%, possibly indicating an effect of the chromosome substitution on the destruction of existing and the formation of novel gene groups with conditionally regulated expression (Figure 6). The preservation rate of overexpressed clusters was the lowest at 0 h (25%) and the highest at 9 h (60%), whereas among underexpressed clusters, it was the lowest at 3 h (36%) and the highest at 16 h (53%). This result was in agreement with the proportions of up- and downregulated DEGs and with the transcriptome expression level in CS and DS across the time points. This finding also points to a major difference between the lines in transcription activity of genes overexpressed at the end of the dark phase and at the beginning of the light phase of the photoperiod. Regardless of the size of clusters, the highest proportions of DEGs were obtained at 0 h in overexpressed clusters and at 16 h in underexpressed clusters, in both lines. Furthermore, in both lines, across the time points, these proportions gradually decreased in overexpressed clusters but increased in underexpressed clusters. The proportion of nonpreserved genes from overexpressed CS clusters was found to be higher in underexpressed DS clusters, whereas nonpreserved genes from underexpressed CS clusters were found mainly in overexpressed DS clusters, but in any case, these proportions were low (up to 14%), possibly indicating the loss or at least weakening of time point–associated expression for most of nonpreserved genes rather than an alteration of this association.

**Figure 7:**
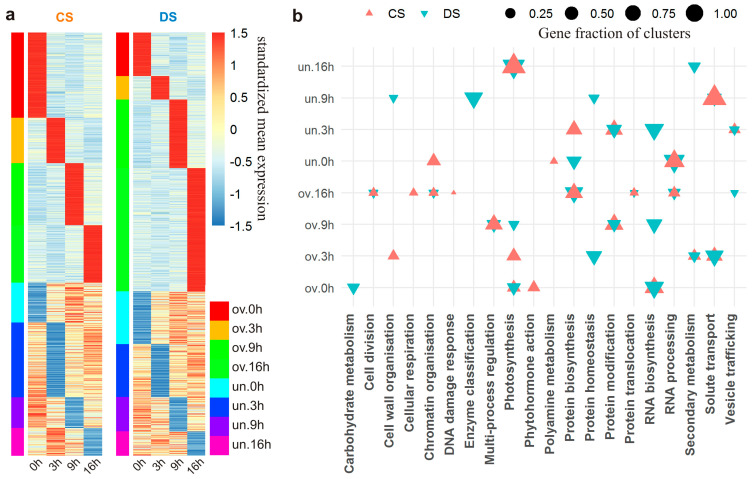
Time point-associated expression. (**a**) Diurnal transcription activity of genes from clusters featuring time point-associated expression [overexpressed (ov) or underexpressed (un) at a certain time point]. (**b**) Over-represented biological processes (*p* < 0.05) in these clusters as a proportion of cluster size.

As follows from the profiles of standardized expression of the time point-related gene clusters (Figure 7a), there were a lot of genes (441 and 356 in CS and DS, respectively) with both a strong positive and strong negative change in activity at different time points (overexpressed at one point but underexpressed at another). Although these pairs of points with opposite changes of gene activities were similar between the two lines, they differed in size. The most abundant were clusters overexpressed at 9 h but underexpressed at 0 h (ov.9 h|un.0 h) and vice versa (ov.0 h|un.9 h) in both lines (Figure 7a). In DS, pairs ov.3 h|un.16 h (*p* < 4.2 × 10^−4^) and ov.0 h|un.3 h (*p* < 7.9 × 10^−7^) were under-represented. Among BPs associated with these genes, “Photosynthesis” was over-represented in CS in both cases (*p* < 1.7 × 10^−3^ and *p* < 2 × 10^−7^). The pair ov.16 h|un.9 h was under-represented in CS (*p* < 1.9 × 10^−6^). These data are consistent with the distribution of up- and downregulated DEGs across the time points, thereby confirming the weakening of expression of “Photosynthesis”-related genes before and at the beginning of the photoperiod (0 h and 3 h) in the substituted line.

The difference in over-represented BPs between the lines was more pronounced for the light phase-related but not dark phase-related clusters (Figure 7b). In the cluster overexpressed at 0 h, “RNA biosynthesis” and “Photosynthesis” were over-represented in both lines. Dark phase-related BPs such as “RNA processing”, “Protein biosynthesis”, “Protein translocation”, “Amino acid metabolism”, “Cellular respiration”, and “Cell division” were over-represented in the cluster overexpressed at 16 h in both lines. Because no common BPs were over-represented among clusters overexpressed at time points 0 h and 16 h, the absence of consolidation in BPs expressed in the middle and end of the dark phase of the photoperiod was assumed. In the cluster overexpressed at 3 h, “Solute transport” and “Secondary metabolism” were over-represented in both lines. “Photosynthesis” was among these processes only in CS, whereas in DS, this BP was over-represented at the next, 9 h, time point. The genes involved in “RNA processing” and “Protein biosynthesis”, which were overexpressed in the middle of the dark phase, were strongly downregulated at 0 h; i.e., at the end of the dark phase of the photoperiod. Strong underexpression was also confirmed for “Vesicle trafficking” at 3 h, “Solute transport” at 9 h, and “Photosynthesis” at 16 h in both lines.

The obtained results were consistent with the transcriptome expression level in CS and DS, just as over-representation of up- and downregulated DEGs across time points, thus implying general weakening of transcription activity in the substituted line before and at the beginning of the photoperiod (0 h and 3 h) with a gradual increase to a peak in the middle of the night (16 h). This includes a shift of the peak of daily transcription activity of such a central plant cell pathway as “Photosynthesis”.

## 3. Discussion

The modern paradigm of divergent evolution of *Triticum* indicates a close relationship between the genome of tetraploid wheat and that of hexaploid wheat. For this reason, substituted lines are often used in wheat breeding to transfer chromosome regions carrying genes of agronomically valuable traits from wild emmer to improved cultivars of common wheat. In the substituted wheat lines, the chromosomes from a different species give compensation to various degrees similarly to different chromosomes of the same species. The specificity of this compensation depends on the evolutionary closeness of species and selective pressure on certain genes. For this reason, similarity between linkage groups from different species, their common occurrence and preservation, or, instead, parallel evolution can be assumed. With respect to genes, this phenomenon reflects expediency (significance) of a given gene set within the same linkage group (chromosome). Despite the high collinearity of syntenic blocks between chromosomes 5B of CS and *T. dicoccoides* (Zavitan), many rearrangements suppress homologous recombination between these chromosomes in some regions of short and long arms [22], implying structural divergence of chromosome 5B. In the present study, functional divergence after the 5B chromosome substitution was also demonstrated in the same substituted line.

Given that germplasm of substituted lines is often incorporated into wheat breeding, it is important to know the genome response of bread wheat to an alien chromosome substitution, in other words, how labile genetic coadaptation of common wheat to accept (compensate) a foreign linkage group of genes (chromosome) is without significant damage to one’s own physiology. Here, it was found that the 5B chromosome substitution from wild emmer was accompanied by changes in transcription activity of at least 7789 genes related to almost all BPs and pathways tested; among them, “Photosynthesis” and “Ribosome” were the over-represented BPs across different tests. Nonetheless, because this is only a small proportion of the reference transcriptome (~14% of genes expressed at a given stage), and taking into account the coregulation (preservation of coexpression) of some DEGs, the physiological compensation of this chromosome substitution seems satisfactory, albeit not completely. In addition, it should be noted that only 20% of the reference transcriptome was analyzed due to the low transcription activity of most genes, whereas the effects of alien chromosome 5B can affect conditionally activated genes in the common wheat genome, which are not expressed in the tested environment and growth stage. Finally, extensive wild emmer gene flow into landraces as compared to cultivars has previously been shown [25]. Because CS is thought to be a Sichuan landrace, a greater degree of genome coadaptation to wild emmer chromosomes is expected more for CS than for cultivated varieties of common wheat. In contrast to the previous findings obtained from genomic data, where there were chromosomes with the largest number of introgressed genomic regions [25], here by means of transcriptomic data, we did not find over- or under-representation of differential gene expression in specific chromosomes, implying a global effect (on the transcriptome) of the chromosome substitution, which perhaps has been implemented through multiple interactions between genes in a cell metabolic network. Thus, local structural changes in the genome (such as the 5B chromosome substitution in this particular case) trigger a global transcriptome response.

A total of 30 BPs and 143 metabolic pathways were tested in the current work with the main focus on “Photosynthesis” as a key BP in the plant cell with predominant activity during the light phase of the photoperiod in both lines. The BPs over-represented in diurnal data on the transcriptome expression level were consistent with plant cell physiology. In particular, “Protein biosynthesis”, “Amino acid metabolism”, “Cellular respiration”, and “Cell division” were among the over-represented BPs upregulated during the dark phase of the photoperiod (7 h after sunset), whereas “Photosynthesis” and related BPs such as “Phytohormone action”, “Solute transport” (at the beginning of the photoperiod), “Secondary metabolism”, and “Carbohydrate metabolism” proved to be upregulated in the light phase of the photoperiod. The main signature of the transcriptome after the 5B chromosome substitution was a sharp significant drop of activity before and at the beginning of the photoperiod.

Secondary metabolites are multifunctional in plant physiology and are primarily involved in defense and interactions with the environment [26]. Here, “Biosynthesis of secondary metabolites” was associated with the largest number of DEGs, thereby pointing to an influence of the wild emmer introgression on responses of wheat to various stressors, as mentioned in the Introduction section. Just like many others, this pathway interacts directly with a key BP of the plant cell: “Photosynthesis”. The latter plays a pivotal role in nitrogen metabolism in leaves, as follows from its interpathway control and signaling [27]. This includes all major stages of mineral nitrogen assimilation, from nitrate reduction [28] to biosynthesis of amino acids and proteins [29]. Accordingly, alteration of the balance of amino acids and enrichment with some of them during photosynthesis creates other conditions for the synthesis of leaf proteins that can have an essential physiological impact. Here, differential activity was documented for many genes of “Nitrogen metabolism”, and in metabolic pathways, almost all proteinogenic amino acids from “Cysteine and methionine metabolism” were most affected. Because “Photosynthesis” was most strongly affected among differentially expressed BPs, it is not surprising that “Carbon metabolism” was also one of the most DEG-rich pathways. In turn, both nitrogen metabolism and carbon metabolism are mediated by modulation of hormones and hormone-signaling pathways [27]. Within the range of DEG abundance (estimated here for the substituted line), the “Plant hormone signal transduction” pathway was closely behind the “Nitrogen metabolism” and “Carbon metabolism” pathways. Because of mutual interactions between different BPs, this short example of interplay between metabolic pathways in the plant cell can be continued until all 143 of them (whose associated genes were present among DEGs) are included.

Although there was no clear epistasis or complementarity after the 5B chromosome substitution, the prevalence of downregulation among DEGs trended toward compositional epistasis [30] between the 5B chromosome of *T. dicoccoides* and the CS genome. This epistasis was not comprehensive and was pronounced only for some genes, and only at certain time points. In particular, although the alien chromosome introgression suppressed the expression of the CS genome to a higher degree as compared to activation (more than a threefold difference) at the beginning of the photoperiod (0 h), this epistatic effect weakened gradually during the experiment, and in the middle of the night, the opposite trend—activation of CS genome expression—was predominant (by almost twofold). Notably, this was true not only for DEGs but also for the tested BPs and pathways. Therefore, complete consistency of results was observed throughout the experiment. Because most of DEGs differed across time points, the effect of the alien chromosome was not permanent; in other words, it did not persist in affected genes. Furthermore, differential activity of chromosome 5B between the lines was comparable in magnitude to that of any other chromosome, with statistical significance only at 0 h. Instead, similarly to all other chromosomes, differential activity of 5B followed a common trend of differential activity of the genome. Consequently, the response of the CS genome to 5B of DS was similar to the response to this introgressed chromosome in the CS genome background. This observation implies a collective, not local, effect on the expression pattern of the CS genome after the 5B chromosome substitution.

A chromosome substitution is also useful for identification of strongly coregulated genes because this approach shows when gene coexpression is preserved during differential expression. Here, it was found that the alien 5B chromosome in the CS genome leads to a loss of coexpression in most cases (75% cases); this phenomenon was accompanied by a drastic change in transcription activity at certain time points or by complete transformation of the expression pattern. Because the numbers of coexpressed genes were similar between the lines, new pairs of coexpressed genes were prevalent in the substituted line. This was also true for the groups of time point–regulated genes. For 421 gene pairs with preserved coexpression, strong coregulation was demonstrated (<25% of common variance in the expression patterns between lines). Nonetheless, it was surprising to find that very few of these coregulated gene pairs consisted of paralogs, suggesting that strong coregulation is not an expected attribute of paralogs. This is because there was less than 50% of common variance of expression between them.

The main limitation of this study is likely the development stage of the analyzed wheat specimens; the third-leaf stage seems to be very early for predicting an effect of an alternative genome response on subsequent growth or for guaranteeing its maintenance after the fifth-leaf stage. Nevertheless, a previously noted delay of the heading of the studied substituted line (15 days) [31] indicates either a permanent or at least an objective impact of the genome response detected at this early developmental stage on the growth rate. The decrease in the growth rate and the delay of heading can be caused by abnormal metabolic activity as well as its predominant suppression in the substituted line. Photosynthesis is the main energy source in the plant cell, and nitrogen metabolism regulation involves almost all physiological processes in plants. Secondary metabolites are also indispensable for plant metabolism and growth. Suppression of ribosome biosynthesis perhaps will affect numerous BPs owing to translation braking. Therefore, the observed downregulation of such pathways as “Photosynthesis” (and associated processes such as “Carbon metabolism”, “Biosynthesis of secondary metabolites”, and “Nitrogen metabolism”) and “Ribosome” in the substituted line seems to be a plausible contributor to the delay of its development and slower growth rates.

Further studies are needed to confirm our predicted alterations in BPs and pathways of common wheat under the influence of the 5B chromosome substitution from wild emmer.

## 4. Materials and Methods

### 4.1. Plant Material and Growth Conditions

Common wheat cv CS and the substitution line of CS with chromosome 5B from *T. dicoccoides* were kindly provided by Prof. B.S. Gill (Kansas State University, Manhattan, KS, USA) and J.D. Faris (USDA-ARS, Fargo, ND, USA). Chromosomal composition of DS was checked by molecular and cytogenetic methods (genotyping, C-banding, and in situ hybridization). The disomic substitution line (DS) was stable and was used in the mapping of chromosome 5B with the F_2_ population of the CS × DS cross and the obtained population of recombinant inbred lines [22,31].

Plants were grown for 21 days after germination under controlled conditions in a Rubarth Apparate climatic chamber (RUMED GmbH) with short days (9 h of light, 20 °C). Three biological replicates of each genotype were harvested into liquid nitrogen at four time points during 24 h since the beginning of the light phase of the photoperiod (0, 3, 9 and 16 h). Time point “0 h” corresponds to the beginning of the light phase (dawn), time point “3 h” is near the middle of the light phase, time point “9 h” matches the end of the light phase (sunset), and time point “16 h” represents the midpoint of the dark phase.

### 4.2. RNA-Seq Library Construction and Sequencing

RNA was extracted using the Plant RNA MiniPrep Kit (Zymo Research, Irvine, CA, USA), followed by DNase treatment with the RNase-Free DNase Set (QIAGEN, Hilden, Germany). Sequencing libraries were prepared by means of the TruSeq Stranded mRNA LT Sample Prep Kit (Illumina, San Diego, CA, USA). Library quality was determined on a 2100 Bioanalyzer (Agilent Technologies, Santa Clara, CA, USA). Sequencing was performed via 75 bp single-end reads on an Illumina NextSeq 550 platform (Illumina) at the SB RAS Genomics Core Facility.

### 4.3. Read Mapping and Counting

A reference transcriptome was obtained from a *T. aestivum* cultivar CS (CS42, accession Dv418) genome assembly (GenBank assembly: GCA_002220415.3) based on annotation of predicted gene models [32,33]. Both high- and low-confidence genes were used. Each transcript was a superposition of all isoforms including 300 bp flanking regions of start and stop codons. Transcripts were extracted from the genome assembly with *gffread* [34]. Single-end sequenced libraries were preprocessed, adaptor and primer sequences were discarded, and the reads were trimmed by quality (no less than 10 bp from the 5′ and 3′ end had to have quality >3), and filtered by length (≥30 bp). The reads were mapped to the reference transcriptome in bwa-mem [35], and mappings with the highest score were utilized for each read. Only uniquely and completely mapped reads were counted. Raw abundance of transcripts was estimated for each gene on the basis of the greatest depth of coverage. Across the study sample, normalization was carried out toward the *MscS-5A* gene (Mechanosensitive ion channel, TraesCS5A02G015600, T4063926).

### 4.4. Modeling of Counts

Following a popular motivation [24,36,37,38], read counts of separate genes and their pools by an averaged count were modeled as following a negative binomial (NB) distribution. Assuming that an NBD is a continuous mixture of Poisson distributions where the mixing distribution of the Poisson rate is a gamma function, it can be parameterized with the mean (*μ*) and shape parameter theta (*θ*, “dispersion parameter”). When *θ* is known, the NB (*μ*,*θ*) distribution is a generalized linear model (GLM) [39,40]. Here, the NB2 model, implying a quadratic function for the mean–variance relationship (*σ*^2^ = *μ* + *μ*^2^/*θ*) was used. Nonetheless, while the mean is given, *θ* is unknown. Although this unknown variable can be estimated during maximum likelihood fitting of an NBD to a set of counts or by the method of moments [41,42], it is unreliable to obtain a correct per-gene estimate of dispersion at a small sample size (such as three biological replicates in our case). An often-used solution is the pooling of information across genes. For this purpose, the counts from genes with similar average normalized counts ≥8 (calculated by the method of moments and rounded) were pooled into bins. To each bin of size ≥30, the NBD was fitted, and parameter theta was estimated with the *fitdist* function of the *fitdistrplus* R package [43]. Despite the popular practice, we did not assume the similarity of count variance among genes of similar average expression or that it can be correctly estimated from the pooled variances. Instead, we evaluated the true observed dispersion across all genes of similar average counts irrespective of the actually unknown expression rate of these genes and variance of their counts. This is essential in the test for significance in the NB GLM, where this dispersion, perhaps specifically for the current experiment, parameterized the H0 (null hypothesis) distribution of counts at a given mean. Predicted values of *θ* for a continuous range of the mean values were calculated from fitted gamma log linear regression of the maximum likelihood estimates of *θ* toward the logarithm of their mean count (bins) using the *glm* function of the *stats* R package.

### 4.5. Adjusting the Mean Count Values and Trimming of Outliers

For each gene, the mean count was estimated iteratively by maximizing the lowest cumulative density probability of a count in an NBD with a current mean and corresponding theta. The counts whose probabilities at maximal lowest probability were less than 0.05 (which are outside 95% area under the probability mass function) were filtered out as outliers, and the mean count was re-estimated for remaining counts until their quantity was >1.0. In case of three replicates, only one outlier can be filtered out, and therefore genes with more outliers were excluded from the analysis. Obtained mean values were repeated two or three times, depending on the number of outliers, to achieve the correct number of degrees of freedom (DFs). Because in the NB2 model, variance is estimated via the mean and parameter *θ* only, this algorithm will give the correct computation of *p*-values in a GLM with categorical predictors (NB regression where gene expression values are the outcome variable and the specimen status [DS/CS, case/control] is the predictor variable), without a need for a range of counts to estimate the mean and DFs.

### 4.6. Differential Expression

The Wald test was performed to identify DEGs. The test statistic derived from this Wald approximation shows how much of standard error of an NBD (with the mean across tested counts and corresponding theta) is included in the difference between the log-transformed tested means. Then, the two-tailed z-test was carried out to calculate a *p*-value (probability) of H0 that logarithmic fold change between means is exactly zero. For this purpose, one NB GLM was fitted for each gene using the *glm* function and the *neg.bin* family object with a logarithmic link (MASS R package) [42] utilizing estimated *θ* for the average of tested counts. The *p*-values were obtained with the help of the summary function with parameter “dispersion” = 1 and were adjusted for multiple testing by the Benjamini–Hochberg correction [44]. Genes with *p*-values < 0.05 were chosen. The fold change difference in gene expression was the base 2 logarithm (log_2_FC) of the ratio of estimated means. Only genes that had ≥8 counts in one of the samples and had absolute value of log_2_FC ≥ 0.9 were kept for differential-expression analysis. To include the genes expressed only in one of lines while avoiding overestimation of the fold change, mean values below eight were raised to eight.

### 4.7. Overall Transcriptome Activity Analysis

Transcription dynamics across time points were evaluated via empirical distributions based on the Kolmogorov–Smirnov test [45], using estimated mean counts as the measure of gene expression. Only genes with the estimated mean count ≥8 at least in one of the samples were chosen. Because the version of the Kolmogorov–Smirnov statistic that has a sign is intrinsically unsuitable for inferring an overall difference between distributions [46], a cumulative shift (the sum of signed differences between distributions) was used as the measure of the difference in transcriptome activity between samples. Furthermore, because the samples were large, asymptotic *p*-values were extremely low, and the test produced statistically significant false positives. To overcome this shortcoming, exact *p*-values were calculated through the permutation test under randomly permutated sample labels. The *p*-value was the proportion of 10,000 resampled unsigned cumulative shifts exceeding the observed one (two-sided test). *p*-values below 1 × 10^−4^ were raised to 1 × 10^−4^.

The variance of gene expression at each time point was estimated via the average squared deviation of expression at a tested time point from the mean diurnal expression of each gene. The logarithmic estimated mean counts as the measure of gene expression were utilized. Only genes with the estimated mean count ≥8 at least in 1 of the samples were used, and the mean count values below 8 were raised to 8. The cumulative shift of the deviation distribution between specimens was computed by the Kolmogorov–Smirnov test [45] as described above, by means of unsigned deviations from the mean diurnal expression of each gene and using the log-transformed counts. Exact *p*-values (significance) of the cumulative shift were calculated by the permutation test as described above.

### 4.8. Correlation Analysis

Pearson’s correlation coefficient was computed from simulated counts. The counts were randomly generated with the *rnbinom* function [47] of the *stats* R package via estimated values of the mean and corresponding theta for parameterization of an NBD. A total of 30 random counts were obtained for each mean and then log-transformed. The correlation coefficient (*r*) was the geometric mean across the coefficients calculated for 16 combinations of 4 independent simulations for each variable. Assuming a normal distribution of the correlation coefficients (because of the central limit theorem), asymptotic *p*-values for *r* > 0.5 at 120 observations at 4 time points (30 observations per point) were < 1 × 10^−5^ (power 0.9).

At time points, the correlation was estimated via simulated data generated as described above, with the help of partial means across time points for each gene.

### 4.9. A Test for Time Point-Associated Expression

In this test, the NB GLM was fitted for each gene by regressing its mean expression at a tested point against its mean expression at each of the other points, utilizing estimated *θ* for the mean of tested counts across all points and DFs as described above. With four time points, this procedure produces three *p*-values for significance of differential expression between a tested point and each of the other points. *p*-values of the genes showing at least a twofold difference in expression between a tested point and each of 3 other time points were subjected to the Benjamini–Hochberg correction [44], and the *p*-value cutoff for each gene was set to 0.05.

### 4.10. Differential Activity of Biological Processes (BPs) and Pathways

Only BPs and pathways containing no less than 100 genes expressed at ≥8 counts at least at one time point were tested. The level of BP activity at a given time point was obtained by the Kolmogorov–Smirnov test [45] as a signed cumulative shift of distributions of expression levels of genes in a tested BP at a given point and those at three other time points. The estimated mean counts were utilized to assess gene expression. The obtained activities indicated over- or underexpression of a tested BP at a given time point relative to its expression at other time points. The significance of this activation was assessed using exact *p*-values estimated with the permutation test. In this test, the null distribution was obtained via calculation of 10,000 values of the Kolmogorov–Smirnov cumulative shift under randomly permutated sample labels. The test *p*-value was the proportion of cases where the resampled signed cumulative shift exceeded the observed one (one-sided test). *p*-values below 1 × 10^−4^ were raised to 1 × 10^−4^. The differential activity of BPs and pathways between the lines was assessed at each time point via the cumulative shift derived from the Kolmogorov–Smirnov test for distributions of the counts for genes in a given BP or pathway at a tested point in DS and CS. Exact *p*-values were computed with the permutation test as described above. Because the exact *p*-values were discrete and came from one-sided tests, the correction for the false discovery rate was not applied.

### 4.11. Functional Annotations

These annotations were assigned to predicted proteins in Mercator4 (v.5.0) [48] and KEGG [49]. In the latter case, gene IDs of the reference transcriptome were assigned to those of KEGG via the mapping of predicted proteins of the reference transcriptome to predicted proteins of “Genome assembly IWGSC CS RefSeq v2.1” (GenBank assembly: GCF_018294505.1) using Diamond (v.2.1.8) [50].

### 4.12. Other Methods

To identify paralogous (including homeologous) genes, predicted protein sequences translated with *gt-seqtranslate* (GenomeTools v.1.5.10) [51] were clustered in CD-HIT [52] at sequence similarity ≥80%. In paralog enrichment analysis, the results were adjusted for the number of genes with at least one paralog in a tested gene set.

The hypotheses of over-representation were tested with Fisher’s exact test [53], and the obtained *p*-values were adjusted for the false discovery rate [44]. Then, the results were filtered out at a *p*-value cutoff of ≥0.05.

A standardized expression level was calculated for each gene from their centered and scaled counts across time points.

## Data Availability

All data necessary to replicate this study’s results are included in this published article (and its Supplementary Materials). Raw data are available upon request.

## Supplementary Materials

The following supporting information can be downloaded at: https://www.mdpi.com/article/10.3390/plants13111514/s1, Table S1: The list of differentially expressed genes (DEGs) in the substituted line at each time point; Table S2: Biological processes (BPs) associated with DEGs; Table S3: Test for over-representation of BPs in DEG sets; Table S4: Pathways associated with DEGs; Table S5: Test for over-representation of pathways in DEG sets.
